# Neural network mapping of gelastic behavior in children with hypothalamus hamartoma

**DOI:** 10.1007/s12519-023-00763-1

**Published:** 2023-11-08

**Authors:** Zhi-Hao Guo, Jian-Guo Zhang, Xiao-Qiu Shao, Wen-Han Hu, Lin Sang, Zhong Zheng, Chao Zhang, Xiu Wang, Chun-De Li, Jia-Jie Mo, Kai Zhang

**Affiliations:** 1 Department of Neurosurgery, Beijing Tiantan Hospital, Capital Medical University, No. 119 South 4th Ring West Road, Fengtai District, Beijing, 100070 China; 2 Department of Neurosurgery, Beijing Neurosurgical Institute, Capital Medical University, Beijing, China; 3 Department of Neurology, Beijing Tiantan Hospital, Capital Medical University, Beijing, China; 4 Department of Neurosurgery, Beijing Fengtai Hospital, Beijing, China

**Keywords:** Contrasted trajectories inference, Gelastic seizure, Hypothalamus hamartoma, Lesion network-symptom mapping, Neural basis

## Abstract

**Background:**

Hypothalamus hamartomas (HHs) are rare, congenital, tumor-like, and nonprogressive malformations resulting in drug-resistant epilepsy, mainly affecting children. Gelastic seizures (GS) are an early hallmark of epilepsy with HH. The aim of this study was to explore the disease progression and the underlying physiopathological mechanisms of pathological laughter in HH.

**Methods:**

We obtained clinical information and metabolic images of 56 HH patients and utilized ictal semiology evaluation to stratify the specimens into GS-only, GS-plus, and no-GS subgroups and then applied contrasted trajectories inference (cTI) to calculate the pseudotime value and evaluate GS progression. Ordinal logistic regression was performed to identify neuroimaging-clinical predictors of GS, and then voxelwise lesion network-symptom mapping (LNSM) was applied to explore GS-associated brain regions.

**Results:**

cTI inferred the specific metabolism trajectories of GS progression and revealed increased complexity from GS to other seizure types. This was further validated via actual disease duration (Pearson *R* = 0.532, *P* = 0.028). Male sex [odds ratio (OR) = 2.611, *P* = 0.013], low age at seizure onset (OR = 0.361, *P* = 0.005), high normalized HH metabolism (OR =  − 1.971, *P* = 0.037) and severe seizure burden (OR =  − 0.006, *P* = 0.032) were significant neuroimaging clinical predictors. LNSM revealed that the dysfunctional cortico-subcortico-cerebellar network of GS and the somatosensory cortex (S1) represented a negative correlation.

**Conclusions:**

This study sheds light on the clinical characteristics and progression of GS in children with HH. We identified distinct subtypes of GS and demonstrated the involvement of specific brain regions at the cortical–subcortical–cerebellar level. These valuable results contribute to our understanding of the neural correlates of GS.

**Supplementary Information:**

The online version contains supplementary material available at 10.1007/s12519-023-00763-1.

## Introduction

Hypothalamus hamartomas (HHs) are rare, congenital, tumor-like, and nonprogressive malformations composed of heterotopic neurons and glia surrounding the hypothalamus [1]. The incidence of HH is estimated to be within the range of one per 50,000 to 100,000 individuals in the population [2]. The most common manifestations were seizures in 61% of cases, precocious puberty in 66% of cases, and both in 25% of cases. Seizures in children with HH are often characterized by the emergence of gelastic seizures (GS), which may later progress to other types of seizures, including focal and/or generalized seizures [3]. The typical onset of seizures ranges from the first days of life to adolescence or early adulthood [4]. GS in children with HH is often drug resistant.

GS, first detected by Trousseau as epileptic nature with the representation of compulsive bursts of laughter [5], has now been described as episodes of uncontrollable mirthless laughter [6]. Therefore, the intrinsic epileptogenicity of HH marked it as an ideal disease phenotype in understanding pathological laughter. Pathological laughter refers to recurrent, episodic, and involuntary laughter in an inappropriate social context and incongruent with an internal emotional state [7]. A summary by an expert review indicated that decreased inhibitory influence at the cortical level (somatosensory cortex) results in the disruption of the cortico-pontine-cerebellar circuits, which subsequently lowers the threshold for motor expression of emotion [8]. Unraveling the neural basis of pathological laughter not only sheds light on future neuromodulation treatment, but also prompts an understanding of normal laughter based on the pathological effect. Previous research has postulated three stages of secondary epileptogenesis that may explain the “kindling-like” relationships between the HH and the neocortex or widespread epileptogenesis [9]. To date, however, the symptomatic evolution of GS remains inconclusive.

Positron emission tomography/computed tomography (PET/CT) imaging with 2-deoxy-2-[^18^F] fluoro-d-glucose (2-[^18^F] FDG) is a sensitive diagnostic imaging modality for detecting epilepsy and a useful diagnostic tool in presurgical evaluation [10–13]. Notably, it not only allows for noninvasive quantification of metabolism, but also enables in vivo examination of brain functions. These aspects have made it a powerful technique for the determination and monitoring of disease pathogenesis and progression, respectively [14]. Processing, coupled with a statistical correlation of GS phenotypes with metabolic images, is also important. In the present study, we sought to better understand GS evolution by employing the contrastive trajectory inference (cTI) algorithm, an unsupervised machine learning technique, to disentangle temporal components and map the complex dynamic disease process from high-dimensional cross-sectional data [15]. Furthermore, we utilized lesion-network symptom mapping (LNSM) to analyze the relationship between the HH-related functional connectivity network and GS symptoms on a voxel-by-voxel basis [16], which might enable the discovery of corresponding brain regions for potential therapy.

The main aims of this study were to (1) explore the clinical characteristics of different GS subtypes; (2) illustrate disease progression using a data-driven approach based on high-dimensional clinical and neuroimaging data; and (3) unravel the lesion behavior-associated brain regions/neurocircuits of GS based on metabolic images. These findings are expected to improve our understanding of the origin and evolution of GS and guide future identification of therapeutic targets for the treatment of neuromodulation.

## Methods

### Patient characteristics

**Fig. 1:**
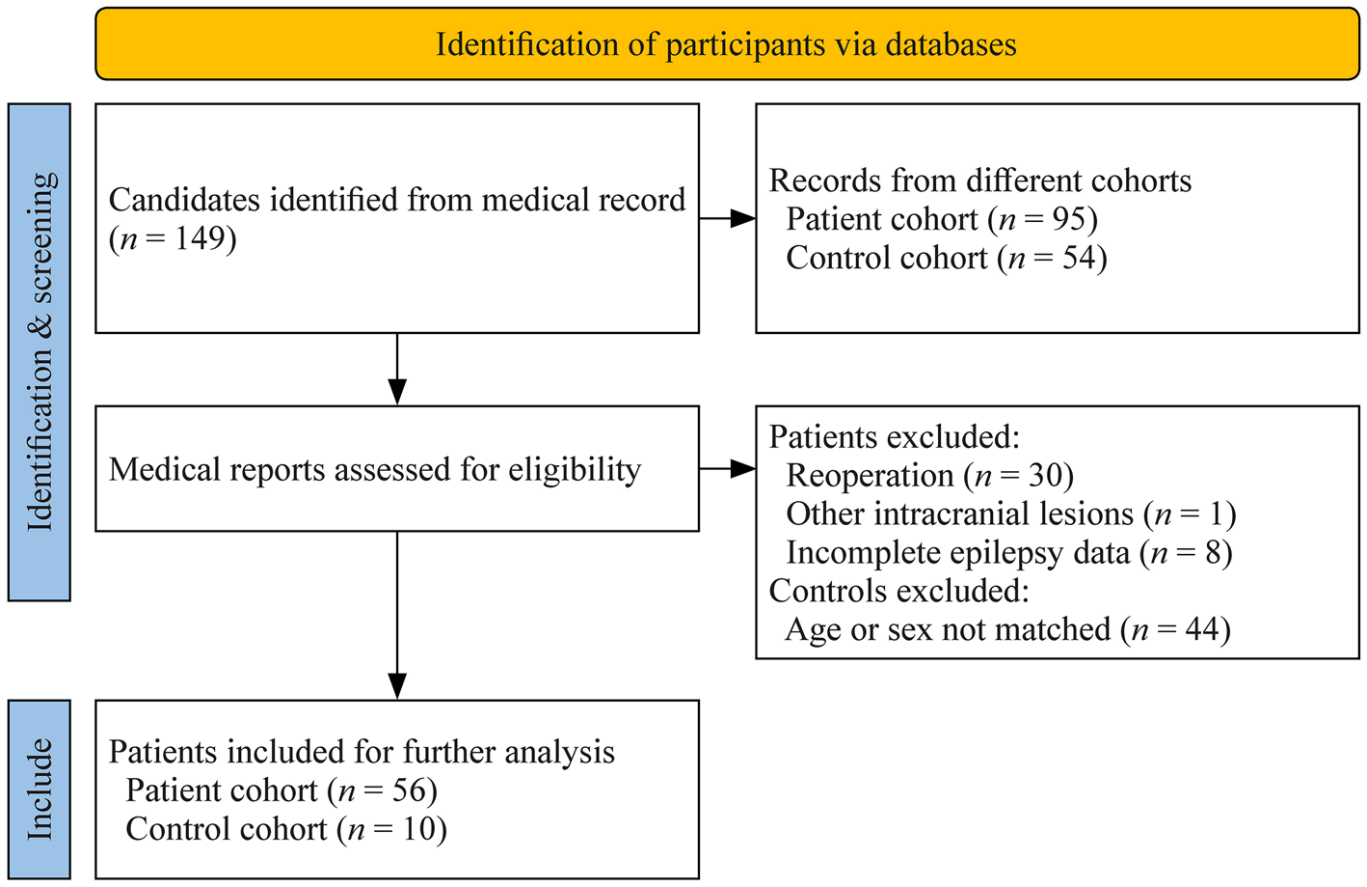
Flowchart of 149 consecutive participants who underwent multidisciplinary presurgical evaluation for epilepsy surgery. Eighty-three participants were excluded due to the exclusion criteria. Finally, 66 participants were enrolled in the study

We screened databases at our hospital for patients who were admitted and treated for drug-resistant epilepsy. Consequently, we selected 56 patients with HH (14 females, age = 8.9 ± 8.2 years, age at onset of epilepsy = 3.0 ± 4.2 years) who underwent multidisciplinary presurgical evaluation. All patients had clinically dedicated imaging investigations, including 3D millimetric resolution structural MRI and ^18^FDG-PET images. Clinical assessment included a comprehensive evaluation of seizure semiology, neurological examination, video-electroencephalogram (EEG) telemetry, and neuroimaging (Fig. 1). We defined the subgroups based on the seizure patterns exhibited by the patients. The definitions of the subgroups are as follows. (1) Te GS-only subgroup includes patients who solely present with GS as their ictal semiology. These patients do not exhibit any other types of seizures. (2) The GS-plus subgroup experiences GS along with other seizure types. These additional seizure types can include focal seizures with impaired awareness, dacrystic seizures, atypical absence seizures, tonic seizures, atonic seizures, secondary generalized tonic‒clonic seizures (sGTCS), epileptic spasms, and so on. (3) The no-GS subgroup comprises patients who do not exhibit GS as part of their seizure presentation. These patients may experience other seizure types, but GS is absent.

### Neuroimaging data acquisition and processing

All participants underwent T1-weighted magnetization prepared rapid acquisition gradient echo (T1WI MPRAGE), T2-weighted fluid-attenuated inversion recovery (T2WI FLAIR), and interictal ^18^FDG-PET, performed using a similar protocol described in our previous studies [17–19].

To map the location and extent of HH, we used the 3D Painting Tool implemented in MRIcron software to draw regions of interest (ROIs) in the anatomical images. In all cases, the final delineation of HH was subject to an experienced expert review. We then warped the native space HH ROI of each patient into standard MNI space.

### Contrasted trajectories inference

Next, we sought to uncover the PET metabolic patterns underlying HH evolution in large-scale biological observations. To this end, we applied recent advancements, the cTI algorithm in the *NeuroPM* toolbox [20], to explore and visualize high-dimensional data. We then calculated mean metabolic values for 166 brain regions, determined based on the Automated Anatomical Labeling atlas 3 (AAL3) [21], which constituted a 56 × 166 matrix for further analysis. We applied contrastive principal component analysis (cPCA) [22], which controls by principal components of the background data to optimize exploration and visualization of the target, for dimensionality reduction and an unsupervised method [23] for preidentifying a selection of features with at least a 0.95 probability of being involved in a trajectory. Each metabolic expression dataset was first adjusted for confounding covariates, namely, age, sex, generalized tonic–clonic seizure (GTCS), volume of HH, and seizure burden. Next, we applied cTI to HH patients and provided the specific trajectories starting on the background data. Each trajectory was composed of the concatenation of a subset of patients, which followed a given pattern in the data’s dimensionally reduced space. Correspondingly, we calculated a metabolism pseudotime value [(0, 1) range], known as the personalized disease index, for each subject. This index reflects the relative position of each subject on the long-term timeline, with relatively low values for subjects with final positions close to the background data and high values for subjects on the distant extremes of the population.

### ^18^FDG-PET image preprocessing

^18^FDG‐PET image data were analyzed voxel by voxel using Statistical Parametric Mapping (SPM12, Wellcome Department of Imaging Neurosciences, London, UK) implemented in MATLAB 2020b (MathWorks Inc, Natick, MA, USA) as previously described [12]. Briefly, the T1-weighted (T_1_w) MPRAGE images were first subjected to nonuniformity correction using the N4 algorithm [24]. Each participant’s ^18^FDG-PET image was registered to their corresponding T_1_w MPRAGE images, and then the Muller-Gartner method was applied to perform partial volume effect correction (PVC) with PETPVC toolbox (https://github.com/UCL/PETPVC) [25]. ^18^FDG‐PET images were spatially normalized into a common Montreal Neurological Institute (MNI) template, and the metabolic value was normalized to the average brain uptake and then scaled to a mean value by “proportional scaling” to reduce individual variation. Thereafter, the PET images were smoothed using an 8 mm isotropic Gaussian kernel to enhance signal to noise over the whole brain. All images were visually inspected to ensure proper preprocessing. We also compared actual disease durations with the pseudotime value across all three subgroups and explored correlations among them using Pearson/Spearman’s coefficients. Subjects were finally stratified into different subtrajectories in the contrasted space [15].

### Voxelwise lesion network-symptom-mapping hypothalamus hamartomas associated with gelastic seizures

We performed voxelwise lesion symptom mapping to determine the relationships between HH and GS [26, 27]. In summary, HH segmentation from each patient was used as a seed in a metabolic connectivity analysis. Consequently, a T map for each patient was created, with each voxel value representing the functional connectivity between that voxel and segmented HH. A threshold was applied for each patient’s T map [voxelwise familywise error (FWE) corrected at *P* < 0.05)] to create a specific mask functionally connected to each patient’s metabolism of HH. A voxelwise one-way analysis of variance (ANOVA), including a between-subjects factor of subgroups (GS-only, GS-plus, no-GS), was used to test whether GS had any significant main effect on the metabolic pattern. To determine the direction of the identified main effects, we performed post hoc groupwise comparisons, with adjustments for the same covariates. All statistical maps were subject to a voxel-level threshold at cluster-based FWE correction (*P*_FWE_ < 0.05) and cluster size > 500. We also applied the Pearson *R* correlation with false discovery rate (FDR) to calculate the intraregional metabolism relationship among subjects in the GS-only subgroup to explore the neural circuit of GS.

### Statistical analysis

All statistical analyses were performed using the SPSS Statistics version 26.0 software program (IBM). Continuous variables are presented as the means (with respective standard deviations) or medians (with their corresponding interquartile range-ranges) as appropriate, while categorical variables are presented as frequencies and percentages. To estimate the relationship between clinical variables and ictal symptoms across the three subgroups, we subjected categorical variables to a Chi-square test, including Pearson Chi-square and likelihood ratio Chi-square tests (for cell frequencies that were less than 5). This was accompanied by symmetric measure tables to strengthen the association between the variables (Cramer’s *V*). Continuous variables were first checked for normal distribution (Lilliefors test) and homogeneity of variances and then subjected to Student’s *t* or Mann‒Whitney *U* tests (for two group comparisons) or ANOVA or nonparametric Kruskal‒Wallis *H* test with post hoc Games–Howell comparisons (for multiple groups). To measure the effect of neuroimaging–clinical variables on GS development, we first performed collinearity diagnostics to exclude multicollinearity [tolerance < 0.1 and variance inflation factors (VIFs) > 10], followed by ordinal logistic regression to identify predictors within each patient subgroup. Next, we applied the Hosmer‒Lemeshow test to test for goodness of fit for the logistic regression model, while Nagelkerke’s *R* squared calculation was conducted to provide an overall indication of the model’s performance. We also computed odds ratios (ORs), 95% confidence intervals (CIs), and *P* values across each group. Statistical significance was represented by *P* < 0.05.

## Results

### Demographic and clinical characteristics of patients with gelastic seizures

Male patients were more likely to experience GS than their female counterparts [*G*^2^(2) = 6.090, *P* = 0.048; Carmer’s *V* = 0.354, *P* = 0.030)], although we found no statistically significant differences in multiple comparison correction. Moreover, we found significant differences between the groups with regard to the age of seizure onset [Kruskal‒Wallis *H* (2) = 11.175, *P* = 0.004], while post hoc Games–Howell comparisons showed that patients in the GS-only subgroup were significantly younger than their no-GS counterparts [mean difference (MD) =  − 8.411, *P* = 0.046]. Patients in the no-GS subgroup experienced significantly fewer seizures [Kruskal‒Wallis *H*(2) = 15.189, *P* = 0.001] than those in the other subgroups (GS-only vs. no-GS, *P* = 0.003; GS-plus vs. no-GS, *P* = 0.007). We found no statistically significant differences in other intragroup parameters, including disease duration, volume of the HH, normalized metabolism of the HH, accompanying symptoms, and follow-up. Patients were compared to ten controls who were matched for age [*G*^2^(3) = 6.994, *P* = 0.072; Cramer’s *V* = 0.339, *P* = 0.055], sex [Mann‒Whitney *U *(1) = 269.000, *P* = 0.884] and scanner. A detailed summary of demographic and clinical data is outlined in Table 1 and Supplementaly Table 1.

**Table 1:** Demographic and clinical data of patients and controls

Variables	GS-only	GS-plus	No-GS	Intra-statistic	Controls	Inter-statistic
Number	17	31	8	/	10	/
Sex (female, %)	3 (17.6%)	6 (19.4%)	5 (62.5%)	***G***^**2**^**(2) = 6.090, *P* = 0.048**	4 (40.0%)	*G*^*2*^(3) = 6.994, *P* = 0.072
				***V* = 0.354, *P* = 0.030**		*V* = 0.339, *P* = 0.055
Age of evaluation (y)	3.0 (2.1, 6.0)	6.5 (2.7, 11.0)	15.5 (11.5, 19.5)	**Kruskal–Wallis *H*(2) = 8455, P = 0.015**	8.5 (6.0,11.0)	Mann–Whitney *U*(1) = 269.000, *P* = 0.884
Disease duration (y)	2.2 (1.5, 4.9)	4.0 (2.1, 9.8)	4.5 (1.2, 9.0)	Kruskal–Wallis *H*(2) = 1.781, *P* = 0.410	/	/
Age of seizure onset (y)	0.5 (0.3, 1.3)	1.5 (0.3, 3.4)	9.0 (4.5, 14.0)	**Kruskal–Wallis *H*(2) = 11.175, *P* = 0.004**	/	/
GTCS (yes, %)	0 (0.0%)	14 (45.2%)	5 (62.5%)	/	/	/
Volume of HH (mm^3^)	180.0 (57.0, 861.0)	160.0 (61.8, 408.8)	70.5 (42.5, 114)	Kruskal–Wallis *H*(2) = 3.389, *P* = 0.184	/	/
Normalized metabolism of HH	1.3 (1.0, 1.5)	1.2 (1.0, 1.3)	1.1 (1.0, 1.3)	Kruskal–Wallis *H*(2) = 3.140, *P* = 0.208	/	/
Endocrinologic dysfunction (*n* %)	1 (5.9%) GHD	1 (3.2%) HOS	2 (25%) CPP	/	/	/
	2 (11.8%) CPP	4 (12.9%) CPP				
Neurocognitive sequelae (*n* %)	2 (11.8%)	3 (9.7%)	0 (0.0%)	*G*^*2*^(2) = 1.672, *P* = 0.434	/	/
				*V* = 0.132, *P* = 0.615		
Intelligent decline (*n* %)	4 (23.5%)	15 (48.4%)	1 (12.5%)	*G*^*2*^(2) = 5.475, *P* = 0.065	/	/
				*V* = 0.303, *P* = 0.076		
Memory decline (*n* %)	4 (23.5%)	14 (45.2%)	5 (62.5%)	*G*^*2*^(2) = 4.018, *P* = 0.134	/	/
				*V* = 0.264, *P* = 0.143		
Seizure burden (times/mon)	150.0 (90.0, 300.0)	75.0 (31.5, 101.3)	18.5 (4.0, 52.3)	**Kruskal–Wallis *H*(2) = 15.189, *P* = 0.001**	/	/
Treatment (*n* %)	4 (23.5%) RFTC	5 (48.4%) RFTC	4 (50.0%) RFTC	/	/	/
	13 (76.5%) LITT	23 (74.2%) LITT	2 (25.0%) LITT			
		3 (9.7%) medication	2 (25.0%) medication			
Follow-up (mon)	12.0 (8.8, 24.0)	12.0 (9.0, 19.8)	17.0 (13.5, 52.5)	Kruskal–Wallis *H*(2) = 2.760, *P* = 0.250	/	/

Continuous data are presented as median (25th percentile, 75th percentile) and categorical data are presented as the number (percentage). *G*^2^ the effect value of likelihood ratio Chi-square test (cell frequencies less than 5). *V* the effect value of Cramer’s *V* for measuring the association between two nominal variables

*GS* gelastic seizure, *GTCS* generalized tonic–clonic seizure, *HH* hypothalamus hamartoma, *GHD* growth hormone deficiency, *CPP* central precocious puberty, *HOS* hypothalamic obesity syndrome, *RFTC* stereoelectroencephalography-guided radiofrequency thermocoagulation, *LITT* magnetic resonance-guided laser interstitial thermal therapy. Bold font indicates statistical significance

### Relationship between metabolism trajectories and gelastic seizures progression

**Fig. 2:**
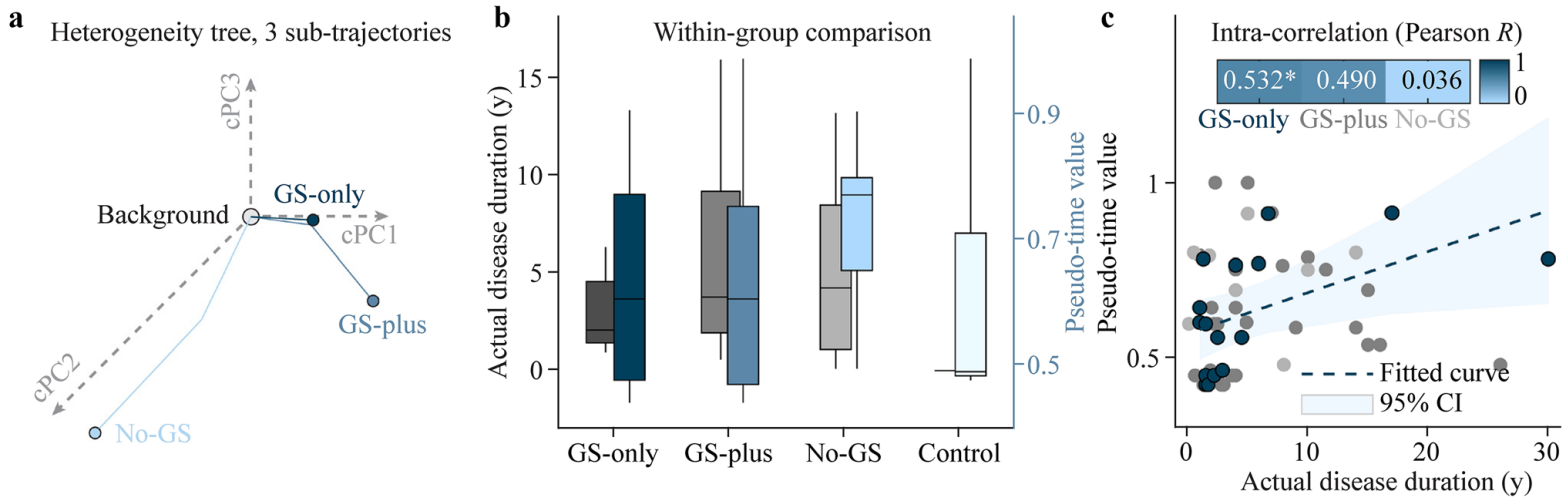
Relationship between disease progression and GS. **a** In the contrasted principal components (cPC) space, each subject is assigned to a PET metabolism trajectory. The subject’s position in the corresponding trajectory reflects the individual’s proximity to the healthy state (the background). An individual pseudotime index of PET metabolism is calculated and reflects the distance to two extremes (background or disease). GS-plus progresses from GS-only, but no-GS has an inverse direction. **b** Horizontal trend comparison of actual disease duration (gray bar) and pseudotime index (blue bar) across the three subgroups. Detailed statistical analysis is provided in Supplementary Table 2. **c** Correlation between actual disease duration and pseudotime index of each subgroup. Blue scatters indicate a significant correlation in the GS-only subgroup. *GS* gelastic seizures, *PET* positron emission tomography, *significant difference

The profiles of the specific metabolism trajectories involved in GS progression are presented in Fig. 2a. In summary, patients in the no-GS subgroup had comparable subtrajectories to the background status. Moreover, patients in the GS-only subgroup had distinct subtrajectories, with a nearly inverse direction, while those in the subtrajectory GS-plus had similar but more extended trajectories than their GS-only counterparts. To illustrate GS progression, we compared actual disease duration with the pseudotime values, calculated by the date-driven method, across the three subgroups (Fig. 2b). The results indicated that patients in the GS-only subgroup had the lowest average age at seizure onset (2.2 years), followed by those in the GS-plus subgroup (4.0 years) and the no-GS subgroup (4.5 years). Disease timelines, based on the cTI algorithm, revealed a similar trend, with patients in the GS-only subgroup recording the lowest pseudotime values (0.46), followed by those in the GS-plus (0.60) no-GS (0.77) subgroups. However, these outcomes were not significantly different [Kruskal‒Wallis *H*(2) = 1.781, *P* = 0.410 and Kruskal‒Wallis *H*(2) = 1.781, *P* = 0.410, respectively] (Supplementary Table 2).

We further explored the relationship between actual disease durations and pseudotime values and found a statistically significant correlation in the GS-only subgroup (Pearson *R* = 0.532, *P* = 0.028) but not in the GS-plus (Pearson *R* = 0.490, *P* = 0.129) and no-GS (Pearson *R* = 0.036, *P* = 0.932) subgroups (Fig. 2c).

### Prediction of neuroimaging–clinical variables for gelastic seizures

**Fig. 3:**
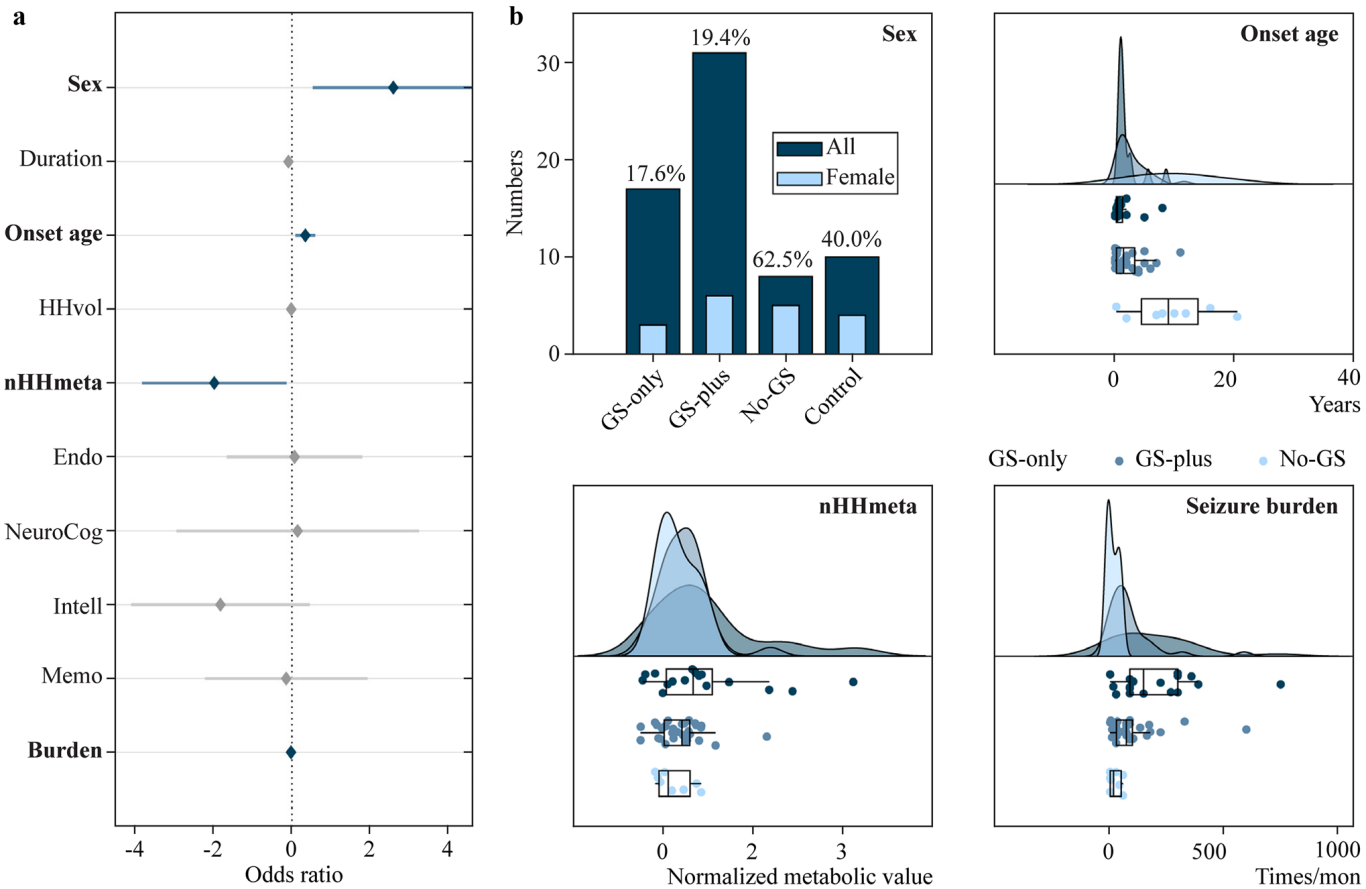
Predictive variables for GS. **a** Forest plot showing the effect of clinical variables on the prediction of GS. Diamonds and the horizontal line represent the individual variable effect (odds ratio) and 95% confidence interval, respectively. When the lines cross the solid vertical line, it indicates no effect and is marked with gray. **b** Bar plot and raincloud plots showing the intragroup comparison of clinical variables. *GS* gelastic seizures, *HH* hypothalamus hamartomas, *onset age* age of seizure onset, *HHvol* volume of hypothalamus hamartoma, *nHHmeta* normalized metabolism of HH, *Endo* endocrinologic dysfunction, *NeruoCog* neurocognitive sequelae, *Intell* intelligence decline, *Memo* memory decline, *burden* seizure burden

The results of collinearity diagnostics revealed that age was a multicollinearity factor (tolerance = 0.072, VIF = 13.906). Considering the issue of multicollinearity, we excluded age from further analysis. Similarly, we did not include the presence or absence of sGTCS, as it can affect patient grouping. Additionally, we did not consider follow-up as a potential predictor in our analysis. The results from ordinal logistic regression indicated that the model had a good fit (*χ*^2^ = 75.659, *P* = 0.967), based on the Hosmer‒Lemeshow test with Nagelkerke *R*-square = 0.617. In summary, this model indicated that male sex (OR = 2.611, 95% CI = 0.551–4.670, *P* = 0.013), younger age at seizure onset (OR = 0.361, 95% CI = 0.107–0.616, *P* = 0.005), higher normalized HH metabolism (OR =  − 1.971, 95% CI =  − 3.821 to − 0.122, *P* = 0.037) and more severe seizure burden (OR =  − 0.006, 95% CI =  − 0.012 to − 0.001, *P* = 0.032) were significant predictors for GS-only HH patients. A detailed profile of the effect of neuroimaging–clinical variables and the intragroup comparison are shown in Fig. 3.

### Voxelwise lesion symptom mapping of HH associated with gelastic seizures

**Fig. 4:**
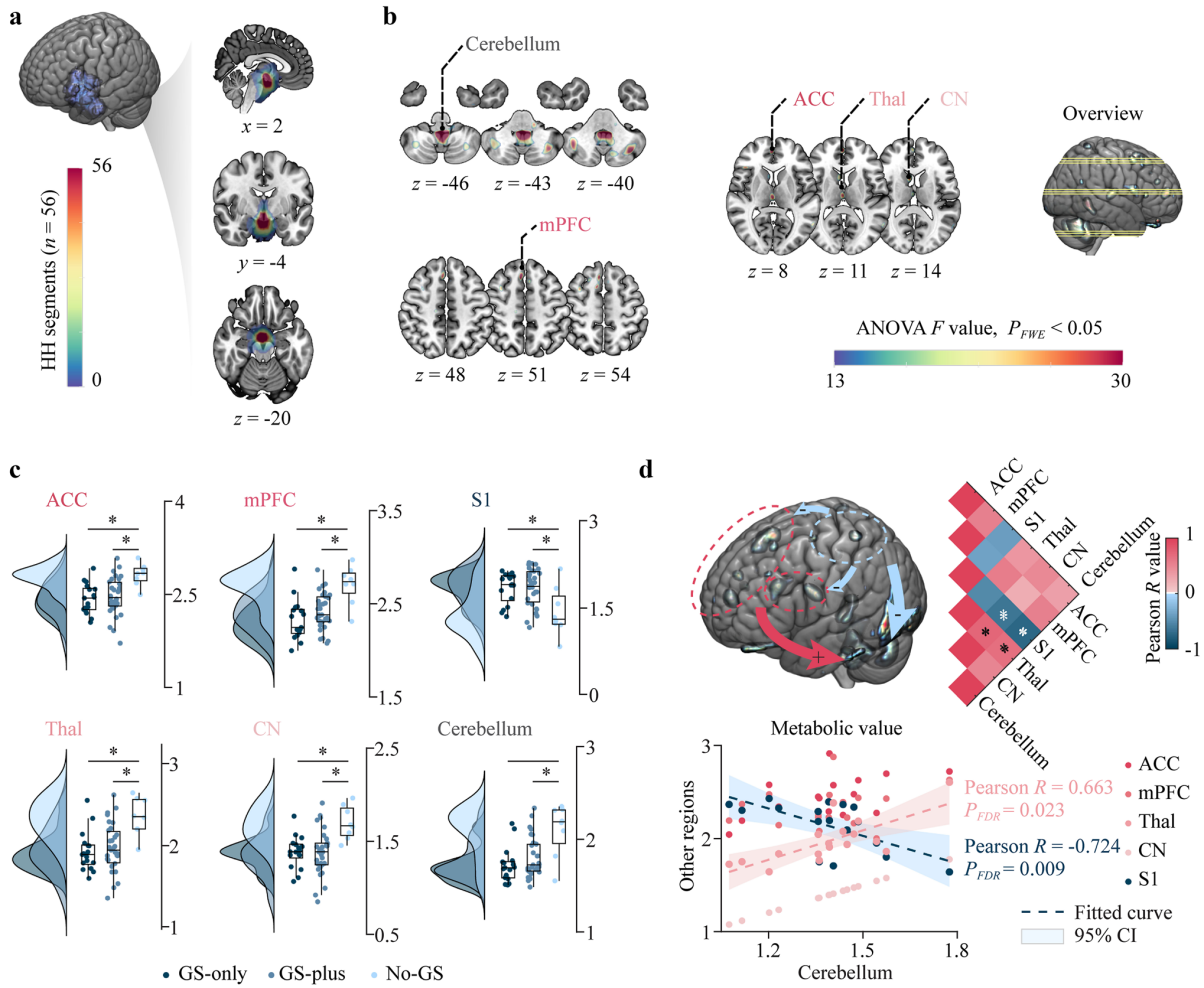
Voxelwise lesion symptom mapping of HH associated with GS. **a** Overlap of individual HH segment maps superimposed on an averaged group-specific template, ranging from an HH volume in a single patient (blue) to overlap in all patients (red). **b** Group-level statistical parametric map displaying the metabolic connectivity difference between GS-only, GS-plus, and no-GS subgroups. The major significant clusters were the anterior cingulate cortex (ACC), medial prefrontal cortex (mPFC), somatosensory cortex (S1), caudate nucleus (CN), thalamus (Thal), and cerebellum. The values are expressed as ANOVA *F* values corrected for multiple comparisons using random field theory at *P*_FWE_ < 0.05 (cluster size > 500). All slices are presented in Supplementary Fig. 1. **c** Within-group comparison of the significant cluster. Detailed statistical analysis is provided in Supplementary Table 3. **d** In the GS-only subgroup, scatter plot analysis showed the metabolic correlation between other regions and the cerebellum. Only the thalamus was significantly positively correlated with the cerebellum (red dotted line), and S1 had a significantly negative correlation (blue dotted line). Detailed statistical analysis is provided in Supplementary Table 4. The matrix plot shows the intraregional correlation. GS gelastic seizures, *HH* hypothalamus hamartomas, *ANOVA* analysis of variance, *CI* confidence interval, *a significant difference

An overview of the extent of HH is presented in Fig. 4a. In summary, group-level statistical parametric maps revealed differences in metabolic connectivity among patients in the GS-only, GS-plus, and no-GS subgroups. Major significant clusters included the anterior cingulate cortex (ACC) (ANOVA *F* = 18.03), mesial prefrontal cortex (mPFC) (ANOVA *F* = 19.11), parietal lobe (PL) (ANOVA *F* = 18.98), thalamus (thal) (ANOVA *F* = 16.67), caudate nucleus (CN) (ANOVA *F* = 16.14), and cerebellum (ANOVA *F* = 25.40). These were obtained at the threshold of *P*_FWE_ < 0.05 and cluster size > 500 (Fig. 4b and Supplementary Figure 1). We then compared differences in metabolic values across the three subgroups and found that all the regions, except PL, exhibited significantly more obvious hypometabolism in the GS-only and GS-plus subgroups than in the no-GS subgroup. Detailed statistical outcomes are presented in Fig. 4c and Table S3. Next, we constructed a GS-associated network targeting GS-pure patients only and found that the frontal lobe (ACC and mPFC) and subcortical structures (thalamus and CN) were positively correlated with the cerebellum. Conversely, the somatosensory cortex (S1) was negatively correlated with the cerebellum as well as the frontal lobe and subcortical structures. Specifically, the thalamus had a significant positive correlation with the cerebellum (Pearson *R* = 0.663, *P*_FDR_ = 0.023), while S1 exhibited a significantly negative correlation with the cerebellum (Pearson *R* = −0.724,* P*_FDR_ = 0.009) (Fig. 4d and Supplementary Table 4).

## Discussion

### Highlights

In the present study, we found that male sex, younger age of seizure onset, higher metabolic value, and seizure burden were predictors of GS. Moreover, we found that GS progression was associated with disease duration and the actual stage of GS evolution. The HH functional network-GS-associated metabolic pattern was identified at the cortical–subcortical–cerebellar level. These results showed the involved circuit and progression nature of GS and revealed potential therapeutic targets for future application as neuromodulation treatments.

### Clinical presentation of gelastic seizures

We investigated the occurrence of GS in patients with HH, which are rare, deep-seated developmental lesions with diverse clinical manifestations. GS is a distinct seizure type associated with HH. GS related to HH initially manifests as focal or partial seizures and can progress to generalized seizures. The presence of GSs is linked to the inherent epileptogenicity of HHs, and their subsequent evolution appears to involve secondary epileptogenesis [28]. The clinical manifestations of GS provide insights into their potential progression, which helps to elucidate the hierarchical grouping observed in our study.

Analysis of the baseline data revealed significant differences in sex, age of evaluation, age of seizure onset, and seizure burden among groups. There are few reports about the association of sex with HH or GS. Li reported that males accounted for 60.3% (129/214) of all patients with HH in a large cohort [29]. The proportions of endocrinologic dysfunction, neurocognitive sequelae, intelligence decline, and memory decline were not significantly different across the three groups in the present study. A study found that among HH patients, 66% experienced precocious puberty, with 25% having both GS and precocious puberty [30]. The underlying mechanism may involve disruption of the hypothalamic–pituitary–gonadal axis by the hamartoma, leading to the early activation of reproductive hormones. Some studies have reported cognitive deficits, including intellectual disability, learning difficulties, and attention deficits, in HH patients with or without GS [31, 32]. However, it is important to note that not all HH patients exhibit cognitive impairment, and the severity and specific cognitive domains affected can vary among individuals. Factors such as the location and size of the hamartoma, as well as the presence of comorbidities, may contribute to the cognitive outcomes in HH patients [33]. The most severe seizure burden was observed in the GS-only subgroup, probably because HHs are intrinsically epileptogenic based on the direct evidence from the intracranial recording electrodes [34]. In clinical practice, additional seizure types are often identified with a clear delay following the start of GS [35], which might be associated with the formation of secondary epileptogenesis. At this time, the ictal epileptic network may be altered and driven by its seizure pattern.

### Inferring the metabolism trajectories of gelastic seizures

In this study, we generated a pseudotemporal trajectory inference field to model the dynamics of metabolism, focusing on static time points. This approach was more effective in illustrating the disease progress of GS. This data-driven method revealed two distinct specific patterns of GS: one is the GS-related pattern with a short pseudotime value of the GS-only subgroup, followed by a progressive branch of the GS-plus subgroup; another is the no-GS subgroup that showed a completely different path and direction. This indicated that the disease trajectory of GS-plus might be developed from that of GS-only subgroup. Pathological laughter in GS is believed to arise due to decreased inhibitory control at the cortical level, specifically in the somatosensory cortex [36], which is also confirmed by our results. This disruption in inhibitory mechanisms can result in the release of motor expression of emotion, leading to the characteristic laughter seen in GS. The symptomatic evolution of GS remains an area of ongoing research. The disease duration was shortest in the GS-only subgroup, followed by the GS-plus and no-GS subgroups, which was related to progressive brain network alterations [30]. In the validation analysis, we confirmed the predictive value of the pseudotime value generated by the cTI method, which had similar trends with actual disease duration. The significant correlation shown in Fig. 2c was observed only in the GS-only subgroup but not in the other two subgroups. The secondary seizure types or epileptic focus might complicate the trajectory. More seizure subtypes of HH should be explored. In summary, our results provide evidence for kindling-like secondary epileptogenesis [9, 37].

### HH-related epileptic network gelastic seizures mapping

Traditionally, regional brain function has been explored by studying deficits that result from neurodegenerative disease or focal brain injury. In this study, we provide a different approach for examining the functional role of neural excitability. Julian proposed a two-hit model of pathological laughter and crying (PLC), which contains direct lesion and indirect diaschisis effects [38]. Another study reported that PLC was associated with widespread dysfunction in the cortico-limbic–subcortico-thalamo-ponto-cerebellar network [37]. Our study mainly focused on GS only and showed a relatively limited network, which involved the frontal lobe, S1, subcortical structures, and brainstem, as well as the negative modulation function of the somatosensory cortex. These results are in line with those reported by functional MRI studies [8, 38, 39]. The cerebellum plays a role in regulating the execution of laughter in cognitive and situational contexts [7]. A recent study further showed that it functions as the “gate control” for emotional expression. It was proposed that cerebellar dysfunction indicated the “disinhibition” or “release” of primitive behaviors, such as crying and laughter.

The identification of specific brain regions implicated in GS at the cortical–subcortical–cerebellar level holds immense promise for future therapeutic interventions and management strategies. These findings establish a solid neuroanatomical foundation for the precise targeting of these regions in future therapeutic approaches. By gaining a deeper understanding of the neural circuits involved in GS, we can pave the way for the development of more focused and efficacious neuromodulation techniques with the potential to significantly improve treatment outcomes.

Despite the significant findings of our study, there are several limitations that should be acknowledged. First, the sample size of our study was relatively small, which may limit the generalizability of our results. Future studies with larger cohorts of patients with HH are needed to validate and extend our findings. Additionally, our study focused primarily on GS and did not fully explore other components of laughter and mirth in HH patients [40]. Further investigations incorporating different components of laughter, neuroimaging representation, genetic phenotypes, and lesion locations are warranted to provide a comprehensive understanding of the complex emotional manifestations in HH.

In conclusion, this study sheds light on the clinical characteristics and progression of GS in children with HH. We identified distinct subtypes of GS and demonstrated the involvement of specific brain regions at the cortical-subcortical-cerebellar level. These valuable results contribute to our understanding of the neural correlates of GS.

### Supplementary Information

Below is the link to the electronic supplementary material.

Supplementary file1 (DOCX 453 KB)

## Data Availability

The corresponding author had access to all data in the study. Anonymized data are available from the corresponding author upon reasonable request.
