# Supplementary material for: Neural network mapping of gelastic behavior in children with hypothalamus hamartoma
Source: World J Pediatr. 2023 Nov 8;20(7):735–45. doi: 10.1007/s12519-023-00763-1 (PMC11269438; doi:10.1007/s12519-023-00763-1)
Supplement: Supplementary file 1 — Supplementary file1 (DOCX 453 KB) [file 12519_2023_763_MOESM1_ESM.docx]

**Supplementary materials**

**Table S1.** Detailed statistical analysis of clinical variables among different HH subgroups

**Table S2.** Detailed statistical analysis of pseudo-time values among different HH subgroups

**Table S3.** Detailed statistical analysis of significant clusters among different HH subgroups

**Table S4.** Intra-regions correlations among metabolic values in the GS-only subgroup

**Figure S1.** Whole-brain voxel-wise lesion-symptom mapping HH associated with GS

**Table S1.** Detailed statistical analysis of the clinical variables among different HH subgroups

| **Variables** | **Test for normal distribution** | | | **Test of homogeneity of variances** | | **Within-group comparison** | | **Post hoc test** | | | | |
| --- | --- | --- | --- | --- | --- | --- | --- | --- | --- | --- | --- | --- |
|  | **Subgroups** | **Lilliefors statistic** | **P value** | **Levene**  **Statistic** | **P value** | **Statistic** | **P value** | **MCC** | **Contrast** | **MD** | **P value** | **95% confidence interval** |
| **Age** | GS-only | 0.360 | **<0.001** | 0.119 | 0.888 | Kruskal-Wallis *H*(2) = 8.455 | **0.015** | Games-Howell | GS-only vs. GS-plus | -2.195 | 0.677 | -8.596, 4.207 |
|  | GS-plus | 0.181 | **0.011** |  |  |  |  |  | GS-only vs. no-GS | -8.411 | **0.046** | -16.689, -0.133 |
|  | No-GS | 0.157 | 0.500 |  |  |  |  |  | GS-plus vs. no-GS | -6.217 | 0.100 | -13.537, 1.103 |
| **Disease duration** | GS-only | 0.298 | **<0.001** | 0.202 | 0.818 | Kruskal-Wallis *H*(2) = 1.781 | 0.410 | - | - | - | - | - |
|  | GS-plus | 0.237 | **<0.001** |  |  |  |  | - | - | - | - | - |
|  | No-GS | 0.160 | 0.500 |  |  |  |  | - | - | - | - | - |
| **Age of seizure onset** | GS-only | 0.318 | **<0.001** | 9.901 | **<0.001** | Kruskal-Wallis *H*(2) = 11.175 | **0.004** | Games-Howell | GS-only vs. GS-plus | -0.843 | 0.435 | -2.495, 0.809 |
|  | GS-plus | 0.212 | **0.001** |  |  |  |  |  | GS-only vs. no-GS | -8.086 | **0.027** | -15.124, -1.049 |
|  | No-GS | 0.116 | 0.500 |  |  |  |  |  | GS-plus vs. no-GS | -7.243 | 0.044 | -14.277, -0.210 |
| **Volume of HH** | GS-only | 0.292 | **<0.001** | 3.162 | 0.050 | Kruskal-Wallis *H*(2) = 3.389 | 0.184 | - | - | - | - | - |
|  | GS-plus | 0.306 | **<0.001** |  |  |  |  | - | - | - | - | - |
|  | No-GS | 0.175 | 0.500 |  |  |  |  | - | - | - | - | - |
| **Normalized metabolism of HH** | GS-only | 0.245 | **0.008** | 4.880 | 0.011 | Kruskal-Wallis *H*(2) = 3.140 | 0.208 | - | - | - | - | - |
|  | GS-plus | 0.125 | 0.241 |  |  |  |  | - | - | - | - | - |
|  | No-GS | 0.198 | 0.466 |  |  |  |  | - | - | - | - | - |
| **Seizure burden** | GS-only | 0.183 | 0.132 | 5.507 | **0.007** | Kruskal-Wallis *H*(2) = 15.189 | **0.001** | Games-Howell | GS-only vs. GS-plus | 108.723 | 0.096 | -16.286, 233.732 |
|  | GS-plus | 0.279 | **<0.001** |  |  |  |  |  | GS-only vs. no-GS | 182.842 | **0.003** | 64.484, 301.200 |
|  | No-GS | 0.282 | 0.061 |  |  |  |  |  | GS-plus vs. no-GS | 74.119 | **0.007** | 18.523, 129.715 |
| **Follow-up** | GS-only | 0.212 | **0.040** | 4.102 | **0.022** | Kruskal-Wallis *H*(2) = 2.769 | 0.250 | - | - | - | - | - |
|  | GS-plus | 0.204 | **0.002** |  |  |  |  | - | - | - | - | - |
|  | No-GS | 0.310 | 0.024 |  |  |  |  | - | - | - | - | - |

HH: hypothalamus hamartoma; MCC: multiple comparison correction; MD: mean difference. Bold font indicates statistical significance.

**Table S2.** Detailed statistical analysis of pseudo-time values among different HH subgroups

| **Variables** | **Test for normal distribution** | | | **Test of homogeneity of variances** | | **Within-group comparison** | | **Post hoc test** | | | | |
| --- | --- | --- | --- | --- | --- | --- | --- | --- | --- | --- | --- | --- |
|  | **Subgroups** | **Lilliefors statistic** | **P value** | **Levene**  **Statistic** | **P value** | **Statistic** | **P value** | **MCC** | **Contrast** | **MD** | **P value** | **95% confidence interval** |
| **Pseudo-time values** | GS-only | 0.155 | 0.325 | 4.173 | **0.009** | Kruskal-Wallis *H*(2) = 1.781 | 0.410 | Games-Howell | GS-only vs. GS-plus | -1.352 | 0.920 | -7.174, 4.471 |
|  | GS-plus | 0.167 | **0.028** |  |  |  |  |  | GS-only vs. no-GS | -0.325 | 0.999 | -7.357, 6.707 |
|  | No-GS | 0.192 | 0.500 |  |  |  |  |  | GS-plus vs. no-GS | 5.100 | 0.055 | -0.098, 10.298 |

MCC: multiple comparison correction; MD: mean difference. Bold font indicates statistical significance.

**Table S3.** Detailed statistical analysis of significant clusters among different HH subgroups

| **Variables** | **Test for normal distribution** | | | **Test of homogeneity of variances** | | **Within-group comparison** | | **Post hoc test** | | | | |
| --- | --- | --- | --- | --- | --- | --- | --- | --- | --- | --- | --- | --- |
|  | **Subgroups** | **Lilliefors statistic** | **P value** | **Levene**  **Statistic** | **P value** | **Statistic** | **P value** | **MCC** | **Contrast** | **MD** | **P value** | **95% confidence interval** |
| **ACC** | GS-only | 0.123 | 0.500 | 1.571 | 0.217 | ANOVA *F*(2) = 0.923 | **0.004** | Bonferroni | GS-only vs. GS-plus | -0.039 | 0.897 | -0.247, 0.169 |
|  | GS-plus | 0.079 | 0.500 |  |  |  |  |  | GS-only vs. no-GS | -0.389 | **0.007** | -0.684, -0.093 |
|  | No-GS | 0.160 | 0.500 |  |  |  |  |  | GS-plus vs. no-GS | -0.350 | **0.009** | -0.623, -0.077 |
| **mPFC** | GS-only | 0.173 | 0.189 | 0.117 | 0.890 | ANOVA *F*(2) = 0.802 | **< 0.001** | Games-Howell | GS-only vs. GS-plus | -0.097 | 0.295 | -0.252, 0.058 |
|  | GS-plus | 0.097 | 0.500 |  |  |  |  |  | GS-only vs. no-GS | -0.382 | **< 0.001** | -0.602, -0.162 |
|  | No-GS | 0.260 | 0.117 |  |  |  |  |  | GS-plus vs. no-GS | -0.285 | **0.004** | -0.488, -0.082 |
| **PL** | GS-only | 0.214 | **0.037** | 0.893 | **0**.415 | Kruskal-Wallis *H*(2) = 7.280 | **0.026** | Games-Howell | GS-only vs. GS-plus | -0.008 | 1.000 | -0.246, 0.229 |
|  | GS-plus | 0.110 | 0.412 |  |  |  |  |  | GS-only vs. no-GS | 0.411 | **0.012** | 0.074, 0.749 |
|  | No-GS | 0.218 | 0.315 |  |  |  |  |  | GS-plus vs. no-GS | 0.420 | **0.005** | 0.108, 0.732 |
| **Thal** | GS-only | 0.157 | 0.307 | 0.603 | 0.551 | ANOVA *F*(2) = 1.127 | **0.001** | Bonferroni | GS-only vs. GS-plus | -0.049 | 1.000 | -0.253, 0.154 |
|  | GS-plus | 0.090 | 0.500 |  |  |  |  |  | GS-only vs. no-GS | -0.432 | **0.002** | -0.721, -0.144 |
|  | No-GS | 0.178 | 0.500 |  |  |  |  |  | GS-plus vs. no-GS | -0.383 | **0.002** | -0.650, -0.116 |
| **CN** | GS-only | 0.190 | 0.100 | 1.096 | 0.342 | ANOVA *F*(2) = 0.695 | **0.001** | Bonferroni | GS-only vs. GS-plus | 0.005 | 1.000 | -0.154, 0.165 |
|  | GS-plus | 0.109 | 0.433 |  |  |  |  |  | GS-only vs. no-GS | -0.315 | **0.004** | -0.542, -0.088 |
|  | No-GS | 0.174 | 0.500 |  |  |  |  |  | GS-plus vs. no-GS | -0.320 | **0.001** | -0.530, -0.110 |
| **Cerebellum** | GS-only | 0.215 | **0.035** | 1.154 | 0.323 | Kruskal-Wallis *H*(2) = 0.126 | **0.010** | Games-Howell | GS-only vs. GS-plus | 0.079 | 0.692 | -0.242, 0.083 |
|  | GS-plus | 0.214 | **< 0.001** |  |  |  |  |  | GS-only vs. no-GS | -0.385 | **< 0.001** | -0.616, -0.155 |
|  | No-GS | 0.233 | 0.229 |  |  |  |  |  | GS-plus vs. no-GS | -0.306 | **0.002** | -0.519, -0.093 |

HH: hypothalamus hamartoma; MCC: multiple comparison correction; MD: mean difference; ACC: anterior cingulate cortex; mPFC: mesial prefrontal cortex; PL: parietal lobe; CN: caudate nucleus; thal: thalamus. Bold font indicates statistical significance.

**Table S4.** Intra-regions correlations among metabolic values in the GS-only subgroup

|  | ACC | mPFC | PL | Thal | CN | Cerebellum |
| --- | --- | --- | --- | --- | --- | --- |
| ACC |  | Pearson *R* = 0.553  P_FDR_ = 0.064 | Pearson *R* = -0.389  P_FDR_ = 0.185 | Pearson *R* = 0.392  P_FDR_ = 0.185 | Pearson *R* = 0.483  P_FDR_ = 0.090 | Pearson *R* = 0.316  P_FDR_ = 0.278 |
| mPFC |  |  | Pearson *R* = -0.357  P_FDR_ = 0.221 | Pearson *R* = 0.566  P_FDR_ = 0.064 | Pearson *R* = 0.226  P_FDR_ = 0.459 | Pearson *R* = 0.536  P_FDR_ = 0.068 |
| PL |  |  |  | Pearson *R* = -0.525  P_FDR_ = 0.068 | **Pearson *R* = -0.633**  **P_FDR_ = 0.029** | **Pearson *R* = -0.724**  **P_FDR_ = 0.009** |
| Thal |  |  |  |  | **Pearson *R* = 0.733**  **P_FDR_ = 0.009** | **Pearson *R* = 0.663**  **P_FDR_ = 0.023** |
| CN |  |  |  |  |  | Pearson *R* = 0.530  P_FDR_ = 0.079 |
| Cerebellum |  |  |  |  |  |  |

ACC: anterior cingulate cortex; mPFC: mesial prefrontal cortex; PL: parietal lobe; CN: caudate nucleus; thal: thalamus. Bold font indicates statistical significance.


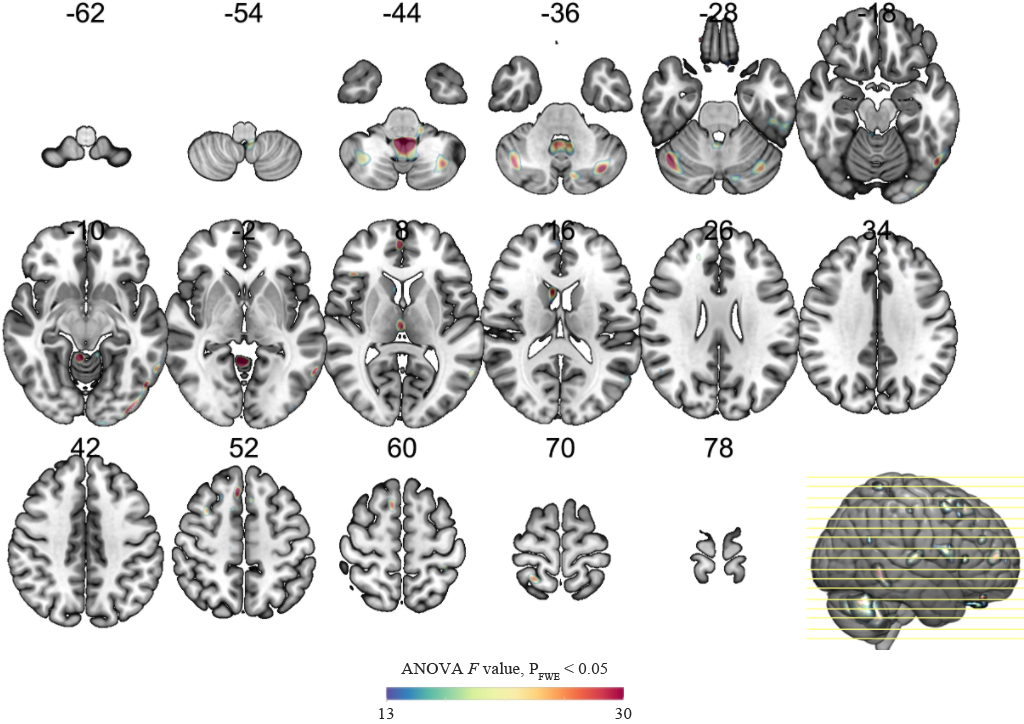


**Figure S1.** Whole-brain voxel-wise lesion-symptom mapping HH associated with GS. Significant clusters were observed in the cerebellum, thalamus, caudate nucleus, anterior cingulate cortex, and medial prefrontal lobe. Color bar represent the voxel-wise ANOVA *F* value (P_FWE_ < 0.05, cluster size > 500 voxels).
